# Supplementary material for: A Multi-Omics Approach to Disclose Metabolic Pathways Impacting Intestinal Permeability in Obese Patients Undergoing Very Low Calorie Ketogenic Diet
Source: Nutrients. 2024 Jun 28;16(13):2079. doi: 10.3390/nu16132079 (PMC11243313; doi:10.3390/nu16132079)

**Supplementary figure S1**: CONSORT flowchart

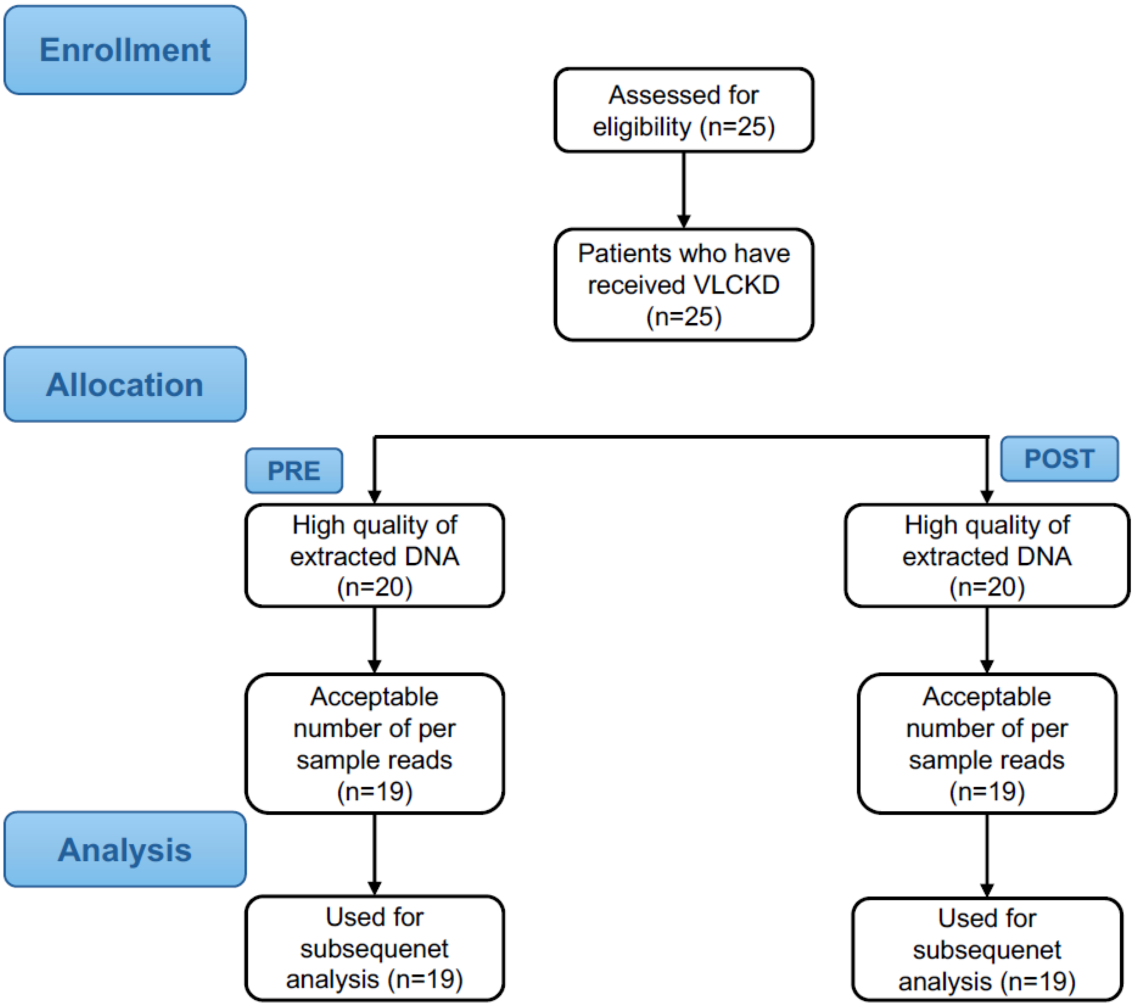

**Supplementary figure S2.** PLS-DA score plot. PLS-DA metataxonomic variables at the genus level were used as input for the PLS-DA. Samples belonging to specific permeability groups was used as stratification criteria. VIP scores are not shown.

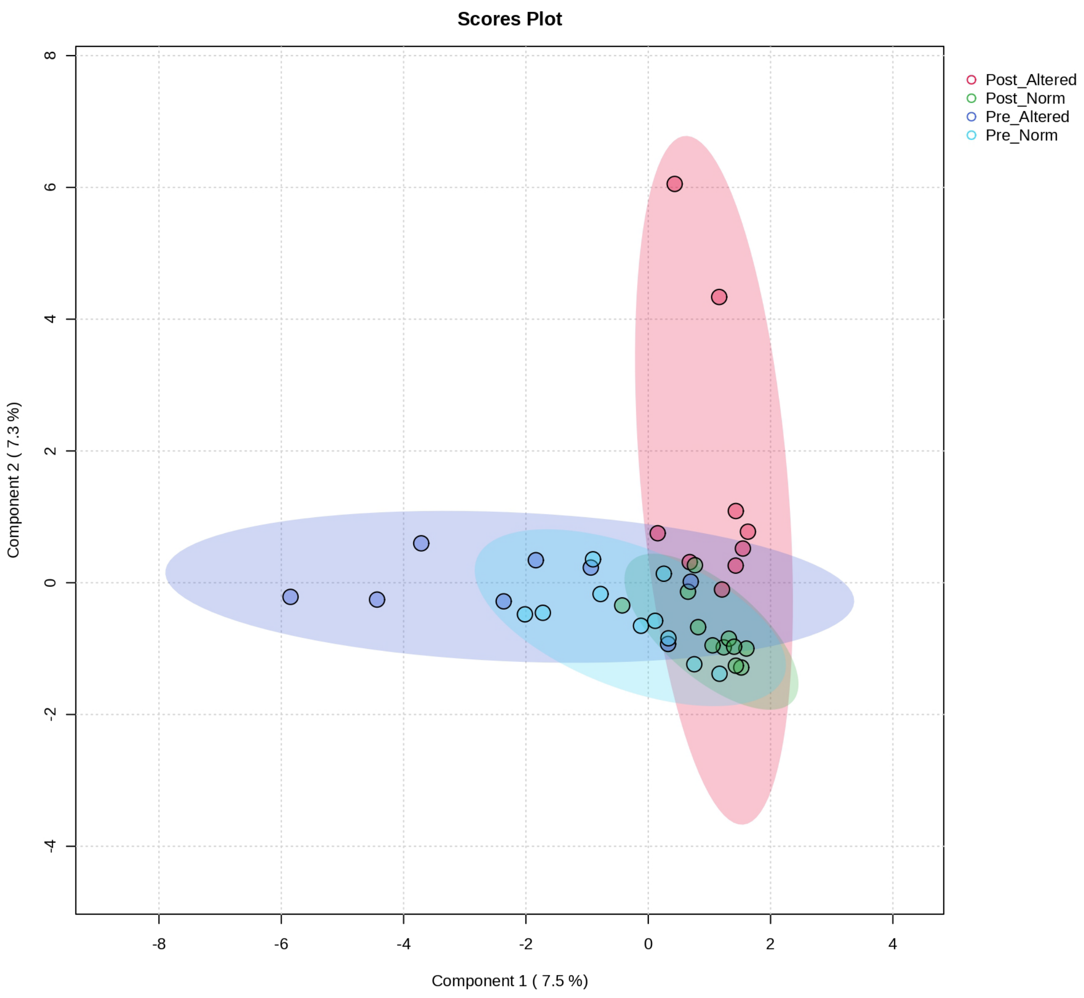

**Supplementary Figure S3**. PLS-DA cross-validation metrics. Cross-validation analysis for each component allowed for estimating Q2 and R2 indices relatively to PLS model accuracy, when working independently of the specific subset used as training set. The goodness of the component accuracy is marked by the red asterisk.

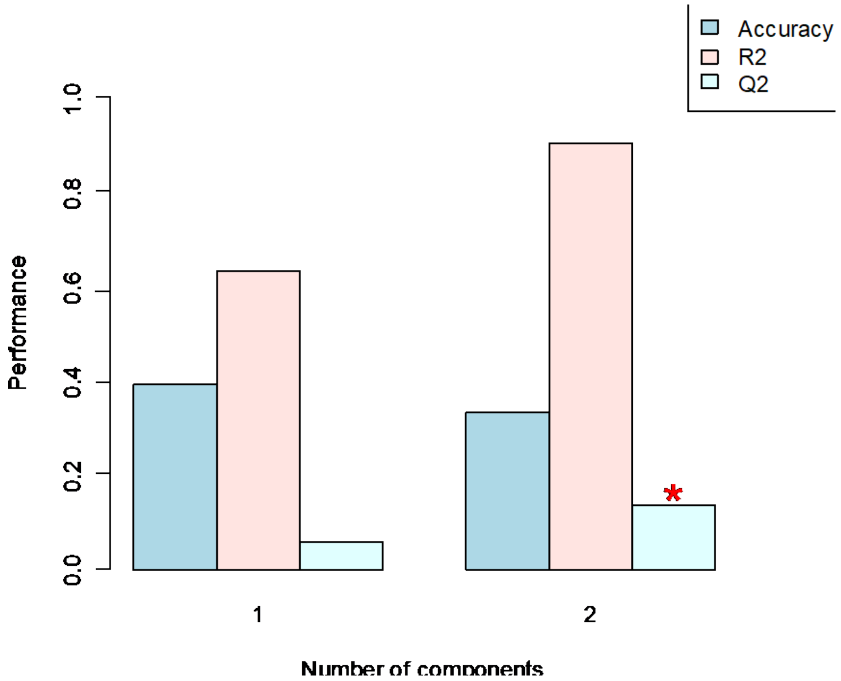

**Supplementary figure S4.** Alpha diversity boxplot. VLCKD treated versus not administered sample boxplot. The sample distribution derived from the application of a Faith's PD has been plotted as boxplot. The table inside the figure reports the output of a Kruskal Wallis paired text and includes the p-value and the corrected p (q-value) obtained after multiple test correction.

| Kruscal_Wallis | | | | |
|---|---|---|---|---|
| Before treatment | After treatment | H | p-value | q-value |
| T1 (n=18) | T2 (n=20) | 6.92307692 | 0.00850904 | 0.02824674 |

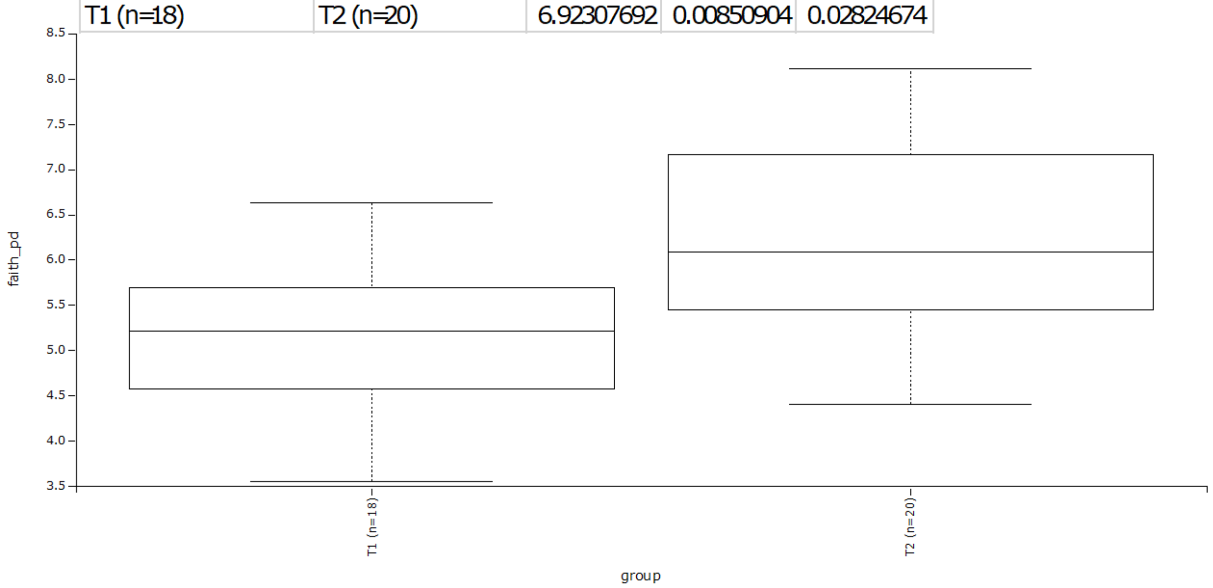

**Supplementary figure S5.** Extended error bar plot of statistically significant bacterial genera that differed as a consequence of VLCKD dietary treatment. Difference in genus mean proportions (95% of confidence intervals) resulted from a Welch pairwise test (corrected by applying a Benjamini Hochberg multiple test) and were reported as error bars for each sample group before (light blue) and after (orange) VLCKD dietary treatment. Because of the direction of the comparison the difference in mean proportion for pre-altered samples appeared as negative values. All the reported genera were statistically significant after correction (q-value < 0.05)

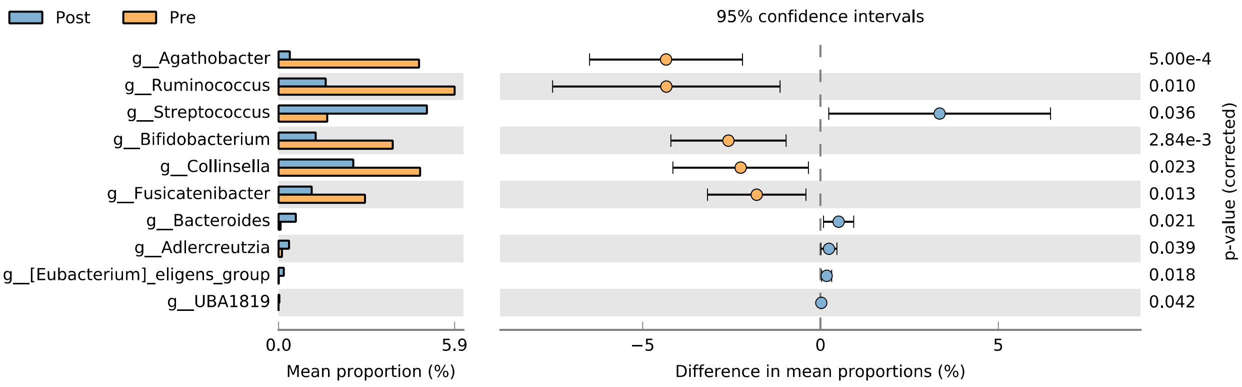

**Supplementary figure S6.** Log2 fold change vs. significance plot in subjects with normal intestinal permeability. Statistically significant Picrust2 pathway obtained by comparing post normal vs. pre normal sample groups have been plotted as a volcano plot indicating increasing and decreasing abundance in predicted pathways, relatively to the VLCKD post normal sample group.

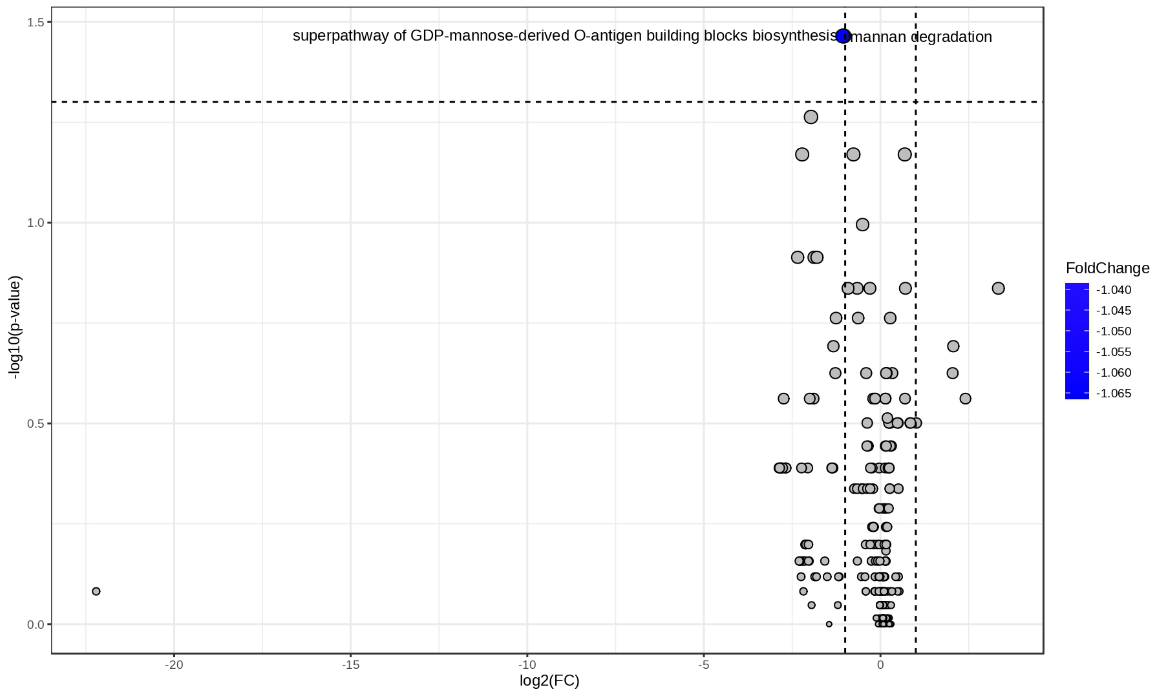

Supplement: Supplementary file 1 [file nutrients-16-02079-s001.zip › nutrients-3023445-supplementary.pdf]
